# Supplementary material for: Blood draw site and blood matrix influence mineral assessment
Source: PLoS One. 2025 Dec 8;20(12):e0338582. doi: 10.1371/journal.pone.0338582 (PMC12685167; doi:10.1371/journal.pone.0338582)
Supplement: S1 Table — This table summarizes the key methodological parameters for the ICP-OES analyses conducted in this study, including the elements assayed, associated wavelengths, and detection limits. It also provides both individual and aggregated quality-control data for internal samples (pooled plasma or serum) and external reference materials (Seronorm). In addition, background concentrations of potential contaminant elements measured across the different blood collection tube (BCT) types are reported. (PDF) [file pone.0338582.s001.pdf]

SUPPLEMENTAL TABLE 1

(1) ICP METHOD DETAILS

element	Ca	Cu	Fe	K	Mg	Na	P	S
wavelength (nm)	373.69	324.754	238.204	766.491	280.27	588.995	213.618	181.972
RF power (kW)	1.2	1.2	1.2	1.2	1.2	1.2	1.2	1.2
plasma flow rate (L/min)	12	12	12	12	12	12	12	12
nebulizer flow rate (L/min)	0.7	0.7	0.7	0.7	0.7	0.7	0.7	0.7
auxiliary flow rate (L/min)	1	1	1	1	1	1	1	1
minimum detection (mg/L)	0.005	0.005	0.005	0.05	0.05	0.05	0.05	0.05
maximum detection (mg/L)	50	5	5	50	50	50	50	50

(2) POOLED PLASMA DATA (mg/L)

element	Ca	Cu	Fe	K	Mg	Na	P	S
sample 1	101.095	1.123	1.996	1243.488	20.176	3102.648	133.066	6864.924
sample 2	103.748	1.164	1.931	1282.903	21.003	3185.959	133.268	7050.922
sample 3	106.440	1.173	1.940	1314.842	21.349	3266.383	136.234	7266.500
sample 4	105.177	1.171	1.910	1305.533	20.998	3244.481	136.363	7239.050
sample 5	105.875	1.173	1.930	1308.389	20.662	3254.619	133.529	7305.229
sample 6	105.257	1.165	1.914	1307.777	21.075	3245.910	133.725	7300.505
sample 7	105.439	1.169	1.925	1310.803	21.086	3248.806	132.848	7387.894
sample 8	104.764	1.182	1.930	1301.810	21.049	3231.686	137.415	7305.305
sample 9	104.720	1.175	1.904	1304.927	20.837	3236.756	132.629	7339.937
sample 10	104.867	1.165	1.900	1298.624	20.826	3226.930	132.782	7392.001
sample 11	99.435	1.114	1.789	1234.913	19.661	3062.788	126.425	7076.540
sample 12	104.041	1.164	1.894	1294.376	20.751	3212.544	128.364	7361.405
sample 13	104.895	1.174	1.927	1306.045	20.982	3240.151	137.186	7487.938
sample 14	102.975	1.144	1.869	1279.253	20.466	3177.831	132.247	7362.154
sample 15	106.079	1.179	1.934	1314.646	21.064	3261.925	133.603	7595.071
sample 16	103.717	1.162	1.876	1286.849	20.593	3197.359	129.226	7526.599
sample 17	104.173	1.165	1.892	1296.085	21.020	3214.179	124.902	7566.395
sample 18	104.895	1.176	1.914	1303.894	20.715	3231.458	125.303	7649.468
sample 19	104.785	1.181	1.902	1306.217	20.756	3235.608	130.354	7636.232
sample 20	105.007	1.166	1.912	1307.527	20.595	3243.677	134.001	7684.627
sample 21	103.401	1.155	1.877	1289.548	20.720	3194.696	131.460	7531.464
sample 22	104.770	1.159	1.900	1308.946	20.834	3242.830	133.910	7718.311
sample 23	105.273	1.170	1.893	1302.947	20.852	3225.829	129.403	7715.306
sample 24	105.416	1.175	1.940	1307.108	20.855	3234.653	128.130	7761.443
sample 25	99.832	1.103	1.838	1239.612	20.005	3077.047	135.829	7349.448
sample 26	104.074	1.157	1.873	1294.332	20.696	3246.703	136.290	7720.964
sample 27	105.623	1.173	1.905	1311.104	20.926	3240.998	132.435	7824.726
sample 28	104.431	1.170	1.891	1302.966	20.741	3223.890	131.121	7755.604
sample 29	102.881	1.154	1.859	1283.152	20.583	3180.013	126.396	7683.446
sample 30	100.078	1.118	1.800	1245.887	19.912	3087.330	125.140	7446.203
mean	104.100	1.161	1.899	1293.000	20.730	3209.000	131.800	7464.000
standard deviation	1.815	0.020	0.041	22.840	0.372	55.630	3.697	235.000
standard error of mean	0.331	0.004	0.008	4.170	0.068	10.160	0.675	42.910
coefficient of variation	1.74%	1.76%	2.17%	1.77%	1.80%	1.73%	2.81%	3.15%

(3) POOLED SERUM DATA (mg/L)

element	Ca	Cu	Fe	K	Mg	Na	P	S
sample 1	95.420	1.172	1.320	191.897	18.908	3132.038	124.199	7455.555
sample 2	100.281	1.223	1.353	201.612	20.144	3280.877	142.572	7897.348
sample 3	100.569	1.228	1.381	203.962	20.357	3312.084	135.430	8003.470
sample 4	100.916	1.239	1.393	202.570	20.165	3288.474	136.089	7972.826
sample 5	99.722	1.215	1.357	201.447	20.225	3280.377	136.341	7937.153
sample 6	95.377	1.180	1.314	192.086	19.135	3182.486	132.890	7582.914
sample 7	100.812	1.231	1.364	203.254	20.121	3298.987	135.929	7995.864
sample 8	100.789	1.240	1.384	202.874	20.416	3352.454	133.853	8063.261
sample 9	100.430	1.226	1.366	203.097	20.150	3307.082	134.443	8043.489
sample 10	92.961	1.147	1.267	187.079	18.527	3059.490	128.521	7464.656
sample 11	95.222	1.166	1.346	189.819	19.018	3148.655	130.896	7539.609
sample 12	98.179	1.204	1.343	198.828	19.887	3279.075	135.167	7920.672

sample 13	99.566	1.224	1.362	202.390	20.196	3323.076	136.402	7996.398
sample 14	100.141	1.231	1.371	202.421	20.052	3333.629	135.808	8056.707
sample 15	100.188	1.234	1.374	202.668	20.381	3297.650	131.428	8100.873
sample 16	95.573	1.177	1.317	191.561	19.255	3135.640	130.072	7748.788
sample 17	98.862	1.214	1.363	199.660	19.841	3286.969	133.575	8039.283
sample 18	99.268	1.210	1.374	200.481	19.948	3250.170	135.448	8078.354
sample 19	100.824	1.231	1.375	203.783	20.315	3297.985	135.274	8230.930
sample 20	98.663	1.218	1.348	199.550	19.714	3232.234	132.761	8070.138
sample 21	104.007	1.241	1.649	199.562	20.586	3293.540	140.066	8485.173
sample 22	104.590	1.243	1.423	200.288	20.788	3301.359	135.298	8520.455
sample 23	103.503	1.239	1.462	198.230	20.738	3266.080	136.319	8466.284
sample 24	105.107	1.245	1.439	200.400	21.288	3314.030	141.285	8593.650
sample 25	101.521	1.217	1.381	192.487	20.300	3187.993	140.472	8270.917
sample 26	105.577	1.259	1.430	201.749	20.880	3298.480	146.768	8585.820
sample 27	110.000	1.298	1.498	210.343	21.956	3436.013	145.446	8927.301
sample 28	106.989	1.272	1.459	204.008	21.500	3358.733	139.412	8741.500
sample 29	106.312	1.271	1.442	203.793	21.217	3341.731	140.508	8704.710
sample 30	101.753	1.211	1.398	191.980	20.308	3167.878	145.208	8318.627
mean	100.800	1.224	1.388	199.500	20.210	3268.000	136.300	8127.000
standard deviation	3.841	0.032	0.069	5.307	0.762	80.450	5.054	378.500
standard error of mean	0.701	0.006	0.013	0.969	0.139	14.690	0.923	69.110
coefficient of variation	3.81%	2.65%	5.01%	2.66%	3.77%	2.46%	3.71%	4.66%

(4) SERONORM DATA - INDIVIDUAL DATA (mg/L)

element	**Ca**	**Cu**	**Fe**	**K**	**Mg**	**Na**	**P**	**S**
**Seronorm Level 1**								
**Day 1**								
replicate 1	91.947	0.986	1.256	122.793	16.555	2695.524	35.360	986.899
replicate 2	88.131	0.973	1.200	120.860	16.137	2660.288	34.807	997.842
replicate 3	85.442	0.940	1.137	116.644	15.408	2569.471	33.400	980.328
**Day 2**								
replicate 1	86.476	0.920	1.275	117.649	16.341	2592.278	39.290	950.993
replicate 2	85.488	0.923	1.208	116.645	16.082	2539.339	39.255	962.982
replicate 3	87.817	0.951	1.225	122.547	16.665	2691.941	41.030	1007.324
**Day 3**								
replicate 1	96.017	0.942	1.507	118.117	16.182	2626.994	42.655	1202.940
replicate 2	88.392	0.932	1.310	116.532	15.859	2581.947	33.538	1185.512
replicate 3	90.617	0.927	1.282	115.929	15.871	2575.554	33.370	1243.050
**Day 4**								
replicate 1	83.457	0.920	1.292	118.253	15.704	2532.525	33.040	*NA*
replicate 2	82.567	0.923	1.271	119.059	15.819	2513.623	33.221	*NA*
replicate 3	84.825	0.953	1.286	122.245	16.185	2581.958	34.092	*NA*
**Day 5**								
replicate 1	83.261	0.927	1.316	116.297	15.718	2585.779	32.780	827.553
replicate 2	85.757	0.930	1.320	117.875	15.764	2605.724	33.355	846.420
replicate 3	90.602	0.983	3.066	123.304	16.371	2710.005	34.629	865.787
**Day 6**								
replicate 1	85.511	0.946	1.286	114.907	15.850	2591.979	34.379	926.128
replicate 2	87.892	0.964	1.275	115.538	16.196	2619.297	35.058	944.176
replicate 3	86.106	0.950	1.245	115.728	16.093	2598.219	35.090	954.928
**Seronorm Level 2**								
**Day 1**								
replicate 1	128.373	1.697	1.745	215.057	32.200	3211.186	94.097	1456.652
replicate 2	136.869	1.711	1.736	217.655	32.690	3235.525	94.882	1476.821
replicate 3	124.018	1.688	1.701	215.139	32.389	3199.745	94.364	1493.714
**Day 2**								
replicate 1	119.471	1.620	1.731	207.722	32.096	3076.609	95.548	1340.842
replicate 2	141.915	1.668	1.807	215.473	33.041	3152.867	99.022	1381.643
replicate 3	165.976	1.691	1.833	217.554	33.992	3177.909	99.836	1401.909
**Day 3**								
replicate 1	134.717	1.614	1.896	206.190	32.329	3090.437	99.570	1845.173
replicate 2	118.316	1.606	1.825	203.367	31.481	3049.710	89.969	1815.908
replicate 3	118.129	1.608	1.844	203.529	31.529	3046.288	89.941	1868.804

**Day 4**								
replicate 1	117.359	1.648	1.845	216.949	32.388	3057.366	95.886	*NA*
replicate 2	120.412	1.719	1.869	223.366	33.072	3173.576	93.986	*NA*
replicate 3	120.171	1.685	1.855	223.565	33.234	3160.290	94.223	*NA*
**Day 5**								
replicate 1	126.388	1.706	1.972	220.643	33.040	3212.548	93.968	1244.522
replicate 2	137.987	1.632	1.865	209.980	31.393	3089.736	89.587	1191.543
replicate 3	123.800	1.702	1.913	221.431	32.973	3219.597	93.960	1255.337
**Day 6**								
replicate 1	121.076	1.679	1.799	208.187	32.656	3161.267	93.501	1356.788
replicate 2	121.920	1.661	1.775	206.960	32.416	3136.586	93.447	1386.108
replicate 3	120.713	1.662	1.783	208.693	32.791	3153.189	93.476	1400.712

(5) SERONORM DATA - SUMMARIZED DATA (mg/L)

element	**Ca**	**Cu**	**Fe**	**K**	**Mg**	**Na**	**P**	**S**
**Seronorm Level 1**								
Day 1	88.507	0.966	1.198	120.099	16.033	2641.761	34.522	988.356
Day 2	86.594	0.931	1.236	118.947	16.363	2607.852	39.858	973.766
Day 3	91.675	0.934	1.366	116.859	15.971	2594.832	36.521	1210.500
Day 4	83.616	0.932	1.283	119.852	15.903	2542.702	33.451	*NA*
Day 5	86.540	0.947	1.917	119.159	15.951	2633.836	33.588	846.587
Day 6	86.503	0.953	1.269	115.391	16.047	2603.165	34.843	941.744
mean	87.239	0.944	1.378	118.385	16.044	2604.025	35.464	992.191
standard deviation	2.678	0.014	0.270	1.860	0.165	35.137	2.420	133.939
**Seronorm Level 1 reference range**								
minimum	69	0.852	1.17	101	13.4	2330	43.3	*NA*
target	86	1.066	1.47	127	16.8	2917	54.2	981
maximum	104	1.281	1.77	153	20.1	3504	65.1	*NA*
**Seronorm Level 2**								
Day 1	129.753	1.699	1.727	215.950	32.426	3215.485	94.447	1475.729
Day 2	142.454	1.660	1.790	213.583	33.043	3135.795	98.136	1374.798
Day 3	123.721	1.610	1.855	204.362	31.780	3062.145	93.160	1843.295
Day 4	119.314	1.684	1.856	221.294	32.898	3130.410	94.698	*NA*
Day 5	129.392	1.680	1.917	217.351	32.468	3173.960	92.505	1230.467
Day 6	121.236	1.667	1.785	207.947	32.621	3150.347	93.475	1381.202
mean	127.645	1.666	1.822	213.414	32.539	3144.691	94.404	1461.098
standard deviation	8.399	0.031	0.067	6.259	0.444	50.985	2.001	230.928
**Seronorm Level 2 reference range**								
minimum	95	1.538	1.72	176	27.1	2820	88	*NA*
target	119	1.925	2.15	221	33.9	3531	110	1335
maximum	143	2.312	2.58	265	40.7	4241	132	*NA*

(7) BCT CONTAMINATION DATA (mg/tube)

element	**Ca**	**Cu**	**Fe**	**K**	**Mg**	**Na**	**P**	**S**
venous plasma BCT	< detection	< detection	< detection	1.987	< detection	2.364	0.272	0.131
venous plasma BCT	< detection	< detection	< detection	2.105	< detection	2.301	0.263	0.209
venous plasma BCT	< detection	< detection	< detection	2.092	< detection	2.316	0.266	0.196
venous plasma BCT	< detection	< detection	< detection	2.092	< detection	2.287	0.263	0.168
venous plasma BCT	< detection	< detection	< detection	2.286	< detection	2.439	0.283	0.224
venous serum BCT	< detection	< detection	< detection	0.169	< detection	2.396	0.269	0.076
venous serum BCT	< detection	< detection	< detection	0.173	< detection	2.452	0.277	0.067
venous serum BCT	< detection	< detection	< detection	0.170	< detection	2.424	0.272	0.064
venous serum BCT	< detection	< detection	< detection	0.174	< detection	2.462	0.279	0.079
venous serum BCT	< detection	< detection	< detection	0.167	< detection	2.391	0.268	0.075
capillary plasma BCT	< detection	< detection	< detection	0.194	< detection	0.002	< detection	< detection
capillary plasma BCT	< detection	< detection	< detection	0.207	< detection	0.002	< detection	< detection
capillary plasma BCT	< detection	< detection	< detection	0.204	< detection	0.002	< detection	< detection
capillary plasma BCT	< detection	< detection	< detection	0.211	< detection	0.002	< detection	< detection
capillary plasma BCT	< detection	< detection	< detection	0.214	< detection	0.002	< detection	< detection
